# A Case Report of Delayed, Severe, Paroxysmal Muscle Cramping After Chilean Rose Tarantula (*Grammostola rosea*) Envenomation

**DOI:** 10.5811/63007

**Published:** 2026-03-10

**Authors:** Luis A. Roque

**Affiliations:** Miller School of Medicine, University of Miami, Miami, Florida. Arácnido Taxonomy, Independent Researcher, Miami, Florida

Dear Editor,

I would like to respectfully bring to the authors’ attention a potentially significant issue concerning the identification of the spider species referenced in the published case report, “A Case Report of Delayed, Severe, Paroxysmal Muscle Cramping after Chilean Rose Tarantula (*Grammostola rosea*) Envenomation.” Upon close examination of the photograph included in the paper, it appears that the specimen may have been misidentified. Based on morphological features visible in the image, the spider in question bears a strong resemblance to *Tliltocatl albopilosus*, rather than the species currently cited.

While I understand that this article is a clinical case report and not primarily a taxonomic study, the accurate identification of the species involved is critical. Misidentification can have meaningful consequences, not only for the broader scientific record but also for the community of arachnid keepers and enthusiasts, as well as for healthcare professionals who rely on correct species identification to assess potential medical risks, treatment strategies, and prognoses in cases involving spider envenomation or exposure.

I therefore urge the authors to consult with a specialist in arachnology or seek confirmation from an authoritative taxonomic source to verify the species designation. Addressing this matter would enhance the scientific rigor of the report and ensure its utility for both clinical and zoological reference.
